# Key Factors That Enable the Pandemic Potential of RNA Viruses and Inter-Species Transmission: A Systematic Review

**DOI:** 10.3390/v13040537

**Published:** 2021-03-24

**Authors:** Santiago Alvarez-Munoz, Nicolas Upegui-Porras, Arlen P. Gomez, Gloria Ramirez-Nieto

**Affiliations:** Microbiology and Epidemiology Research Group, Facultad de Medicina Veterinaria y de Zootecnia, Universidad Nacional de Colombia, Bogotá 111321, Colombia; saalvarezmu@unal.edu.co (S.A.-M.); nupegui@unal.edu.co (N.U.-P.); apgomezr@unal.edu.co (A.P.G.)

**Keywords:** America, RNA virus, virus host interactions, zoonoses

## Abstract

Viruses play a primary role as etiological agents of pandemics worldwide. Although there has been progress in identifying the molecular features of both viruses and hosts, the extent of the impact these and other factors have that contribute to interspecies transmission and their relationship with the emergence of diseases are poorly understood. The objective of this review was to analyze the factors related to the characteristics inherent to RNA viruses accountable for pandemics in the last 20 years which facilitate infection, promote interspecies jump, and assist in the generation of zoonotic infections with pandemic potential. The search resulted in 48 research articles that met the inclusion criteria. Changes adopted by RNA viruses are influenced by environmental and host-related factors, which define their ability to adapt. Population density, host distribution, migration patterns, and the loss of natural habitats, among others, have been associated as factors in the virus–host interaction. This review also included a critical analysis of the Latin American context, considering its diverse and unique social, cultural, and biodiversity characteristics. The scarcity of scientific information is striking, thus, a call to local institutions and governments to invest more resources and efforts to the study of these factors in the region is key.

## 1. Introduction

Throughout the course of human history, we have faced multiple epidemics caused by infectious agents with viruses playing an important role, although the factors that explain the jump between species remain unclear. In the search for answers, aspects associated with the biology of viruses, the ecology of the hosts, and changes in biodiversity have been implicated as determinants in the emergence of diseases with pandemic potential; however, the interactions among these factors are still poorly understood. Thus, it is important to establish what elements favor infection with viral agents that represent a threat of global distribution, whose origin is undetermined, for which there is a highly susceptible human population, and with currently no treatment and/or vaccines to allow their control and/or prevention. Even though there are numerous pathogens of great importance in public health, viruses with an RNA-type genome that belong to the *Coronaviridae* and *Orthomyxoviridae* families are particularly relevant. Therefore, this review will focus on these two families due to their human-to-human transmission capacity, the broad range of hosts they can infect, and their responsibility on some of the latest pandemics of the 20th and 21st centuries.

RNA viruses have compact genomes and a high mutation rate (10^−3^–10^−5^ errors per nucleotide and replication cycle) [1], generating changes in its sequence as a result of errors not corrected by RNA-dependent RNA polymerase or due to recombination processes. These changes can occur as a result of the normal replication cycle or be related to evolutionary mechanisms of viruses [2], involving the processes of mutation, selection, and drift, which, depending on their location and/or function in the viral genome, will have a distinct impact. Taking influenza virus as an example, the changes that lead to differences in pathogenicity are polygenic, thus the understanding of molecular mechanisms and associated factors that make it possible for specific changes to result in adaptation, jump between species and/or increase in pathogenicity is crucial. In this context, the differences in the NP gene related to host ranges are noteworthy since adaptive mutations have been identified in influenza viruses that caused the 1918 (H1N1) and 2009 pandemics (H1N1pdm). In a similar fashion, the PB1 gene, particularly the amino acid in position 375 is associated with a host-range signature. Most avian strains have an asparagine in this position while most human influenza strains have a serine. Interestingly, when analyzing influenza viruses that caused the pandemics of 1918, 1957, and 1968, which contained segments of the PB1 gene of different origin, it was found that in the avian strain asparagine was replaced by a serine at position 375. In addition, the fact that a serine can be found in several avian viruses in this position and that some human H3N2 viruses contain asparagine in the same position [3] raises questions about whether other compensatory mutations are produced either in the PB1 gene or in another viral protein, noting that the mechanism by which mutations occur is yet to be fully understood. Similar to this, it is important to establish the effect that specific changes in the virus can have on the innate immune response by inhibiting the induction of interferon and promoting viral replication. In some cases, a substitution of an amino acid is observed in H5N1 viruses where a change in PB1-F2 N66S could increase its virulence.

Furthermore, recombination homologous or non-homologous is a common attribute of many RNA viruses related to an increase in the rate of adaptation. Another mechanism for variability is the reassortment of viruses with a segmented genome in which, when a single cell is infected by multiple genotypes, the progeny consequently inherits a mixture of gene segments from the parental viruses. These rearrangement events often generate new phenotypes and have been associated with host switching in numerous viruses [4]. This results in dynamic populations where balance and pressure for selection are established between mutations or changes generated and the selected viral populations that will be maintained affecting the susceptible population [5].

The *Coronaviridae* family has been one of the most representative models of viral evolution in nature. With the largest genome among RNA viruses, coronaviruses have been distinguished by their unique changes at the gene level, adopting increasingly efficient forms of transmission broadening their host range while generating a large number of variants thanks to their replication characteristics [6] and their high mutation rate estimated between 2 to 6 × 10^−4^ nucleotides/genome/year. Even though some mutations are usually deleterious, others facilitate transmission and adaptation to new hosts [7]. Furthermore, the co-evolutionary capacity with their hosts has also placed a determining point in the crossing between species.

Regarding the *Orthomyxoviridae* family, the most notable example of rearrangement is the antigenic shift seen in influenza A viruses. The rearrangement among influenza virus strains from mammals and birds could involve the antigenic profile, this defined by the genes that encode for hemagglutinin (HA) and neuraminidase (NA) of one strain with host-specific determinants of another, leading to antigenic shifts [8]. These rearranged viruses represent new antigens for the susceptible population, leading to pandemic spread in the unprotected demographic [9]. The three influenza virus A pandemics that occurred in the 20th century (1918 H1N1, 1957 H2N2, and 1968 H3N2) [10] originated from rearrangements that resulted in the exchange of HA from avian strains with mammalian influenza viruses [11]. However, based on the complete genome sequence of the virus that caused the 1918 pandemic, it has been proposed that, unlike the influenza viruses accountable for the 1957 and 1968 pandemics, it was the result of a direct adaptation to humans from a virus of avian origin [3].

Likewise, in the 21st century, the first pandemic occurred in 2009 and was due to H1Npdm influenza virus, initially detected in pigs, and whose genome resulted from a very particular rearrangement that included influenza A virus genes of avian, swine, and human origins [12]. Thanks to the exponential development of knowledge and the adoption of control measures, it was possible to contain and overcome the pandemic quickly, however, the repercussions in the social and economic sphere were remarkable, ratifying the role of influenza viruses as one of the most important pathogens that could have a dramatic impact on public health and the world economy. This pandemic also served to establish guidelines for interdisciplinary work, training of specialized personnel, along with the development and implementation of infrastructure with a global vision and scope, which served as a baseline and as a model to face the current SARS-CoV-2 pandemic. This is reflected in the strategies implemented for the study and control of COVID-19, being the first pandemic caused by a beta-coronavirus, at a time when advances in molecular biology have allowed in record time the mass-production of information related to the virus, and its biological and molecular characteristics. However, during the making of this review, there are still numerous questions both concerning the virus per se, its origin, pathogenesis, immune response mechanisms, and about the role of other environmental and anthropogenic factors that could have influenced in the appearance of this virus with pandemic characteristics.

The objective of this systematic review was to analyze the factors that favor the generation of infections with pandemic potential focusing on the characteristics of viruses with an RNA genome and their relationship with the host at a global level, with an emphasis on the Latin American context.

## 2. Materials and Methods

### 2.1. Search Strategy and Identification of Relevant Studies

A systematic review was carried out by analyzing the literature submitted to peer-review and published in international databases in English, during the period 2000–2020. The articles were obtained from the Scopus database (https://www-scopus-com, accessed on 7 December 2020) and ScienceDirect (https://www-sciencedirect-com, accessed on 7 December 2020) through the institutional access granted by the National University of Colombia. PubMed databases were also searched. Research articles were included in the search while other types of publications (Review articles, Book chapters, Conference abstracts, Editorials, among others) were excluded.

Using the aforementioned databases, keywords that were found as MeSH terms and Boolean connectors were selected. The “filters” tool was used to refine the search by year (“refine by”), establishing the range of interest (2000–2020) and the type of article based on the inclusion and exclusion criteria described above. MeSH and Boolean terms used were: (Virus–host Interactions) AND (pandemic) AND (virus). MeSH terms such as (viral transmission) AND (RNA virus) AND (America) were also used to select articles that would allow contrasting the information published internationally with that reported in Latin America, data included in this systematic review as a case study. Likewise, in the search equation the MeSH terms (bat OR Chiroptera) AND (avian OR bird) were included considering the epidemiological role that these species have in the spread of viruses with pandemic potential and limiting the search in animal species.

### 2.2. Charting of Data and Statistical Analysis

With the articles obtained through these search strategies, a database was created in Microsoft Excel^®^, with the following fields: article title, author(s), year of publication, journal, country under study, considered species, type of methodology used, number of samples analyzed, type of infection (natural or experimental), changes reported in viruses and their relationship with pathogenicity, ecology and/or behavior of natural hosts, and environmental or anthropogenic factors that favor inter-species transmission. Once the database was completed, the articles were selected using two strategies: they were initially selected based on the title and the abstract, excluding articles in the areas of therapy and vaccination. Subsequently, a critical reading of the article was performed, completing the database items described above and excluding those that did not meet the inclusion criteria. The analysis of the database in Microsoft Excel^®^ was carried out using descriptive statistics, establishing averages, percentages, and frequencies of presentation.

## 3. Results

Out of the 130 articles found in the search based on the above criteria, 61.5% (*n* = 82) were excluded. A total of 48 articles that met the inclusion criteria were analyzed, 18 of which specifically addressed the Latin American context. Regarding the methodology applied in the articles, 14.6% (*n* = 7) used molecular biology techniques, 41.6% (*n* = 20) were bioinformatic studies, 18.8% (*n* = 9) studied the influence of cultural practices involved in the emergence of zoonotic diseases with pandemic potential, and 25% (*n* = 12) performed regression, spatial and univariate analyses. 

Asian countries such as Singapore, Thailand, and China, among others, have been the focus of study due to the usual presence of live animal markets, associated with events such as the emergence of influenza and coronavirus viruses. Remarkably, 8.7% of the articles reviewed were related to China and 6.5% to Australia. Only 2.1% of the articles contained data from African countries such as Kenya, Botswana, and Uganda. Due to the interest of the systematic review in the Latin American case study, the selected studies that reported information on Latin American Countries (LAC) were 16%. 

In countries that have studied factors associated with the jump of viruses between species, 19.5% (*n* = 9) of the authors have mostly related bats as the main reservoirs and disseminators of different types of virus, followed by 10.8% (*n* = 5) that have associated different wild animals. On the other hand, other studies have been performed in different species: 32.6% (*n* = 15) have done so in humans, 8.7% (*n* = 4) in both marine mammals and birds, and in rodents 13% (*n* = 6). This also highlights the participation of other species in the evolution of viruses.

Multiple approaches have been used to understand the factors that impact the transmission of viruses across species. Aspects associated with the biology of the virus, host ecology, and behavior, and changes in global biodiversity seem to be closely related to the emergence of diseases with pandemic potential (Table 1), although the interactions among them are poorly understood. In this systematic review, we present the scientific evidence between 2000 and 2020, mostly from countries affected by prominent sanitary emergencies, demonstrating the interest among investigators to improve disease surveillance systems and control of emerging and reemerging diseases.

### 3.1. Factors Associated with the Virus and its Hosts 

RNA viruses employ a great variety of evolutionary mechanisms to survive and adapt to environmental and host-related conditions. Molecular mechanisms that influence transmissibility, pathogenicity, receptor union, immune response evasion, and cross-species interactions, have attracted great attention within the scientific community [13,14,15,16]. Coronaviruses have adopted ever-evolving effective routes of transmission holding a broad range of hosts. Outstandingly, bats have been well described as natural hosts of a variety of viruses, highlighting their role as amplifiers and disseminators of coronaviruses at a global level. Network analyses made with phylogenetic data have shown that bats have a higher degree of connectivity with a large number of bat viruses, representing a common platform for viral exchange and mixing. Clades of coronavirus found in non-flying mammals have exhibited a relationship with bat-related viruses and show high connectivity. On average, any bat species interacts with nearly two types of viruses, as compared with rodent species where there is nearly one-to-one rate host–virus interaction [17,18]. It has been proposed that genetic drift helps to promote the variability of these viruses and considering the presence of multiple bat species within a colony, the viral jump across species is a highly probable event. 

**Table 1:** Factors that enable pandemic potential of RNA viruses and interspecies transmission.

	Factor	Ref
**Virus**	**Changes in cell membrane binding proteins.** e.g.,: Glycoprotein S mutations in SARS-CoV-2 (D614G of region S1-S2 near furin cleavage site).	[19]
	**Changes in other internal viral proteins.** e.g.,: mutations at central region (intrinsically disordered protein regions—IDRs) of nucleocapsid protein in SARS-CoV-2.	[20]
	**Modification of cell-entry approach by the virus.** e.g.,: HA protein activation in H17 viruses by changing the pH conditions in the cell.	[10]
**Host**	**Receptor recognition and affinity with viral proteins.** e.g.,: affinity between ACE-2 receptor and S protein of SARS-CoV-2. e.g.,: Sialic acid receptor affinity dependent on saccharide ending SAα-2,3 and SAα-2,6-Gal for influenza viruses.	[14,21]
	**Intrinsic factors associated with variation in disease presentation and severity.** e.g.,: host pro-viral genes involved in chromatin remodeling (complex SWI/SNF), histone modification, cellular signaling, and RNA regulation key for development of coronavirus infection.	[22]
	**Host immune-response evasion by alteration of gene expression.** e.g.,: High and Low pathogenic H5N1 induces alternative splicing in genes such as ADAR, CCL19, RIG-I, DDX60, LGP2, MDA5, IRF7, MX1, NLRC5, STAT1, TRIM25, and VIPERIN in ducks.	[23]
	**Host ecology and behavior.** e.g.,: bats and birds have an extraordinary viral richness due to flying capacity, long migrations, trophic diversity, and social structure	[18]
**Other factors**	**Anthropogenic factors:** population density, distribution patterns, traveling and international trading, technological advances in agriculture, changes in farming and food consumption patterns (food source or for medicinal purposes). e.g.,: In Asia, live markets are characterized for the trading of wild animals such as squirrels, rats, porcupines, and wild birds and pigs.	[24]
	**Environmental factors:** loss of biodiversity and of habitat for wildlife, deforestation, and climate change, associated with an increase in wildlife-human interaction rate. e.g.,: Brazilian regions diminishing the migratory behavior of bats, which impacted the viral dynamics of the Hendra virus.	[6]

The circulation of alphacoronavirus in Europe and Asia has been associated with the bat species that acts as the natural reservoir, representing an ideal ecological context for recombinant mediated evolution, recognized as a key phase in the process of host transmission by adjusting and improving the adaptability of a bat virus to a terrestrial mammal [25]. Bats that belong to the genera *Pipistrellus, Eptesicus, Rhinolophus*, and *Hipposideros* hold the alpha and beta coronaviruses, while fruit bats (genera *Pteropodidae*) only host betacoronavirus assigned to Lineage D [17]. This suggests a virus–host specificity relationship in bats, for which the development of new studies that contribute to establish the relationship between endemic bat species and named coronavirus to settle surveillance and control strategies against a possible zoonotic emergence is needed. 

Primates, rodents, and bats seem to hold zoonotic viruses that are not yet well connected to domesticated and other wild species, supporting the premise that these species share zoonotic viruses directly with humans. It has been suggested that bats host a wide variety of viruses while non-human primates serve as amplifiers and disseminators. Due to the genetic similarity with humans, this could further favor the exchange of viruses between humans and primates [26]. Circumstantial findings of bat coronaviruses in other mammal hosts have been extensively called into question. Chen et al. [15] reported that SARS-CoV-2 has a 96% similarity with sequences isolated from bats when analyzing ORF1a, ORF1b, S, ORF3a, E, M, ORF6, ORF7a, ORF7b, ORF8, N, and ORF10 genes, thus the zoonotic implication is highly probable. Although the intermediate host is unknown, there is 85–95% similarity between sequences of SARS-CoV-2 and coronaviruses sampled from pangolins, making them a possible intermediary for human infection [15,27]. However, current information is not conclusive to determine the pangolin as the official intermediate host for this virus [28]. On the other hand, different authors point out that the insertion mutation of an amino acid in position 20–21 could explain the cross-species viral jump for this virus [27].

Changes at a genomic level in coronaviruses reflect their implication in the evolution of these pathogens. Particularly, single nucleotide polymorphisms (SNP) profiles reveal that ORFs that codify for RdRp and S proteins, ORF8, and NP of SARS-CoV-2 have suffered mutations at the host level, evidencing a key point for changes related to transmissibility and virulence. Using high-throughput sequencing (HTS) and transcriptomics data from studies in humans infected with SARS-CoV-2, Ghorbani et al. [29], defined a high-resolution map of the SNP hot spots of the SARS-CoV-2 in a viral population at the inter-host level showing that at least 22 sites have significant differences in mapped readings suggesting potential RNA modifications. The positions of the SNPs in the coding regions of the SARS-CoV-2 ORFs were located in RdRp ORF (14 SNP), S ORF (4 SNP), ORF8 (2 SNP), and N ORF (1 SNP). The Cytosine by Thymine SNP with a variable frequency ranging between 5% and 99.8% was the most abundant SNP, positioned only in regions of the genome corresponding to polyprotein and protein S. Although the true functional impact of low-frequency SNPs at the protein S sequence level remains unclear, mutation 24 positioned in the signal peptide domain and other SNPs located in the S2 domain could modify viral tropism, suggesting that the virus could infect new hosts. More studies are needed to compare enough virus sequences from different geographic locations to better understand the intra-host behavior and selection pressure. The amount of modifications at NSP8 suggests that this could affect its replication rate and if crossing of the host barrier is allowed, its replication would be faster and more efficient [7,19,29,30]. It is reported that some of the most representative homoplasies in the genome of this virus are found at site 11.083 which comprises a region of ORF1a that encodes the non-structural protein Nsp6, and at site 21.575 that corresponds to the spike protein. The great genetic variability of the virus reported in April 2020 by Van Dorp et al. [7] in the UK, the USA, Iceland, and China suggests that each of these epidemics at the local level have been due to multiple independent introductions of the virus. In this sense, each time a SARS-CoV-2 viral particle replicates in a susceptible host, it facilitates the presentation of errors in the generation of new copies of the genome, giving rise to the appearance of variants.

It is important to determine high-frequency SNPs as key components to understand viral evolution principles providing us guidelines for the improvement of control strategies, such as vaccine development. Frequently spotted sites of mutations in the nucleocapsid gene reflect the importance of tracking changes in SARS-CoV-2 due to its involvement in the recognition of epitopes by the immune system. The genes of the nucleocapsid, ORF6, ORF7a, ORF8, and ORF3a were found to be more likely to mutate. However, if we focus on non-synonymous mutations, the protein S genes like ORF1a / b and ORF3a contain the highest number of this type of mutations which could be involved in changes in the functionality of the viral protein. M and E genes are more conserved and have been associated with housekeeping functions within the genome. The relative abundance of a mutation can be taken as an advantage over viral viability, which would be dictated by the effect of the mutation on the stability of the protein and its function with respect to specific biomolecular events during host-pathogen interaction [19]. The SNPs and amino acid substitution observed in quasi-species of SARS-CoV-2 reflect the quasi-species ability to rapidly increase its polymorphism among infected humans during an outbreak and to evade the immune response, increasing transmissibility, generating resistance to antiviral drugs, and affecting the sensitivity of molecular diagnostic tests [19,30]. Thus, the monitoring of accessory genes should also be a priority during vaccine development. In the case of SARS-CoV-2, the ORF10 encoding a presumed ubiquitin ligase is the most expressed accessory gene in the virus [29]. In this sense, the participation of these accessory genes as key players in immune evasion is an important aspect to understand the specific response against the pathogen.

Even though different phylogenetic analyzes have indicated the ancient origin of human coronaviruses from animal relatives, their evolutionary history is yet to be established. However, the sharing of certain types of codons between SARS-CoV-1 and SARS-CoV-2 and the suppression of dinucleotides, show that their origin could be to some extent close [15]. These differences could also be explained by the mutational pressure and selection in genes with different lengths since selection could maximize the efficiency of the translation process of longer energy-cost genes and reduce the size of highly expressed proteins.

Glycoprotein S plays an important role in cell membrane binding through the Angiotensin-converting enzyme 2 receptors (ACE2) of the host. Some of its characteristics have remained similar across different coronaviruses, although certain structural and molecular variations have given relevant advantages in terms of the infection process. The structure of SARS-CoV-2 glycoprotein S is highly similar to the one found in SARS-CoV-1; the difference is evident at the receptor recognition domain conformation and its location [31]. Glycoprotein S mutations such as D614G of region S1–S2 near the furin cleavage site could have important repercussions for cross-species transmission and enhance zoonotic infection for this virus [30]. These mutations could be involved in the adaptability and transmission processes, for example, variants that have low stability of their S protein have a low transmission capacity [19]. Thus, the relative abundance of certain mutations would provide an advantage for viral viability. The union affinity between S protein of SARS-CoV-2 and ACE-2 receptor suggests a broader range of hosts, possibly affecting primates, bovines, hamsters, dogs, cats, pigs, ferrets, pangolins, and others. However, it is key to clarify multiple and additional conditions that are required besides the sole presence of the virus for an infection to develop [14,32]. Even though the changes in Glycoprotein S create variations in the way the virus enters the cell, these are not the only determinants. As an example, changes in other viral proteins such as nucleocapsid protein, seem to participate in the interactions between intrinsically disordered protein regions (IDRs) supporting cross-species transmission. These regions are widely conserved in most coronaviruses with a certain pattern of uniformity between the Alpha, Beta, and Gamma genera. This characteristic has been associated with fecal–oral transmission and to the firmness of the virus capsid. IDRs are also characterized by having regions rich in amino acids such as Arginine and Serine but poor in aromatic amino acids in all coronavirus genera. Although the amount of polar amino acids is low in Beta and Gamma, these are replaced by Lysine. This location aspect can be of importance in the pathogenicity and transmission between hosts, mainly associated with regions rich in Serine for phosphorylation processes. Central IDRs make cardinal contributions to the multitasking role of the nucleocapsid protein, probably requiring structural plasticity, also influencing coronavirus host tropism and interspecies transmission [20].

Similarly, for influenza viruses, receptor recognition due to their affinity for sialic acid receptors is key for interspecies transmission. Two types of receptors have been described based on their binding to saccharide endings, SAα-2,3 and SAα-2,6-Gal. Avian influenza viruses (AIV) bind to SAα-2,3-Gal receptor type, found in the respiratory and gastrointestinal tracts of birds. In turn, human seasonal influenza viruses preferentially bind to receptors of the SAα-2,6-Gal type found in the upper respiratory tract in humans. Nonetheless, SAα-2,3-Gal receptors have been found in the lower respiratory tract of humans, making an eventual avian influenza virus zoonotic transmission possible. Pigs, on the other hand, carry receptors of both types α2,3 and α2,6 acting as an intermediate host in which viruses of avian and human origin can withstand rearrangements and generate viruses with the ability to cross the interspecies barrier. There is also evidence to suggest that other animals, particularly some land bird species such as the quail, could provide an environment similar to pigs by exhibiting both SAα2,3-gal and SAα2,6-gal receptor types in trachea and intestine [21].

Another well-described factor that contributes to the adaptation of viruses to new hosts is their immune response evasion mechanisms. Influenza viruses avoid host immune response through alternative splicing of innate response genes, thus interfering with antiviral reaction. High and Low pathogenic H5N1 induces splicing in genes such as ADAR, CCL19, RIG-I, DDX60, LGP2, MDA5, IRF7, MX1, NLRC5, STAT1, TRIM25, and VIPERIN in ducks. These genes play a fundamental role in the immune response of AIV, but when the virus produces new isoforms of these genes, ducks enter an immunosuppressed state and viral replication is enhanced [23]. Influenza viruses have been characterized for their adaptability and ample host range, associated with the close contact to natural hosts and with constant recombination and reordering of their viral genome. The generation of subtypes with increased pathogenicity have evolved with new ways to enter the cell or to boost hemagglutinin activation, as seen with, for example, H17 viruses able to change the pH conditions inside the cell. A cleaved HA protein can activate its membrane fusion function under low pH conditions, accompanied by enzymatic cleavage and conformational rearrangements. However, H17 shows susceptibility to trypsin at a high pH (8.0), similar to HA proteins cleaved at a low pH, suggesting that H17 may utilize a different mechanism for membrane fusion. For example, instead of using the endocytosis mechanism to activate its protein due to the low pH of the endosome, the fusion could occur on the surface of the cell membrane at a neutral pH. In addition to this, at the receptor binding site in hemagglutinin, tight bonds of hydrogen bridges are formed limiting the usual binding between HAs with the receptor (e.g., the carboxylate groups of the sialic acid negatively charged with both sides of residue 136 chains, usually T or S in all known HA subtypes except H17) [10]. On the other hand, the H5 AIV subtype serves as a good example for spillover, where the introduction of viruses from migratory wild birds to terrestrial birds has facilitated the establishment of lineages and sub-lineages in different regions of the world, thus favoring the possibility of gene exchange contributing to diversity and variability of viruses with pandemic potential.

Gene expression variance among hosts has been proposed to influence pathogenesis and disease severity. The HMGB1 protein is a pleiotropic protein that binds to nucleosomes regulating chromatin in the nucleus, carries genetic material, and acts as a sentinel for non-self-nucleic acids. By participating in the regulation of the expression of ACE2 receptors, the protein responds to SARS-CoV-1, SARS-CoV-2, and NL63 infection. For coronavirus, host pro-viral genes involved in chromatin remodeling, histone modification, cellular signaling, and RNA regulation, along with the different routes of infection could explain the variation in disease presentation and severity caused by SARS-CoV-2. Susceptibility to the disease can be correlated with the expression of host genes that could provide certain resistance. Genes that codify for the chromatin remodeling complex SWI/SNF promote the entrance of the virus into the cell, a process that is also affected by chromatin remodeling associated genes that modify the regulation of the ACE2 receptor [22].

### 3.2. Other Factors That Affect the Interspecies Transmission 

Changes adopted by the virus are influenced by the environmental context and host-related habits and characteristics, which defy the ability of viruses to adapt and survive [33]. Such aspects mostly relate to population density, distribution patterns, host migration, and loss of natural habitats. The increase of the human population as a constituent for globalization, technological advances, and worldwide migration, have led to environmental alterations such as fragmentation, deforestation, and the loss of habitat for wildlife [34,35]. As a consequence, wild animals tend to seek shelter near human settlements, increasing the probability of wildlife-human interaction [6].

**Figure 1:**
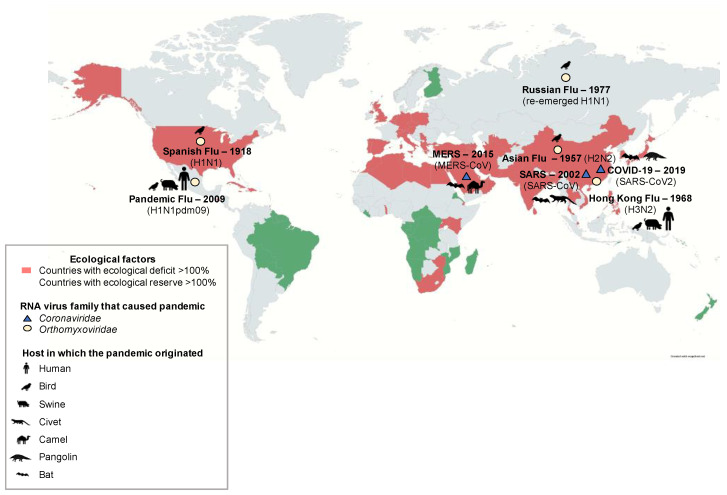
Countries with a percentage greater than 100% of ecological deficit (red) and countries with ecological reserve (green). The map indicates countries of origin of pandemics caused by RNA-genome viruses that belong to *Coronaviridae* (blue triangle) and *Orthomyxoviridae* (yellow circles) families. In the parenthesis are the viruses accountable for pandemics and pictograms represent the host(s) for each one of them. The year refers to the start date of the pandemic. Ecological data for deficit/reserve were taken from: Global Footprint Network, www.footprintnetwork.org (accessed on 7 December 2020).

Furthermore, based on reports presented by the Intergovernmental Science-Policy Platform on Biodiversity and Ecosystem Services (IPBES) in 2019, the extinction rate of wild species has substantially increased having serious repercussions on human health and quality of life at a global level [36]. According to The Global Footprint Network (GNP), the ecological footprint of many countries is above their biocapacity, entering into an ecological deficit, associated with the decrease in the ecological assets of nations and with higher extinction rates [37]. This ecological deficit is also related to the introduction of biocapacity through trade, posing a risk to health across borders. Countries with the highest biocapacity deficit (>150%) are Singapore, Israel, Qatar, the Republic of Korea, and Japan (Figure 1). Even though LAC have a greater reserve of biocapacity, the interaction between humans and wildlife has increased due to the loss of 20% of the Amazon forest in just 50 years. In 2019, the destruction of the Amazonian ecosystem was estimated at 18% [38] with a forest loss up to 2.4 million hectares (24,000 km^2^) in Brazil, Bolivia, and Peru [39]. In Colombia, between 2012 and 2015, a loss of 187,955 hectares due to deforestation and almost double the territory (414,605 hectares) as a consequence of the degradation of the Amazon was reported. Deforestation and degradation processes bring the Pan-Amazonian ecosystem closer to the “tipping point” [40], which implies that some vital processes for the health of populations stop working, favoring the transmission of zoonotic diseases by reducing the “dilution effect” of pathogens in areas of the world with greater biodiversity and therefore greater presence of reservoirs [41,42]. To exemplify this, the loss of wild habitat in the Brazilian Amazonian regions has diminished the migratory behavior of bats, impacting the viral dynamics of the other highly important viruses such as Hendra virus. Since the bat population decreased, the virus crossed the species barrier, and an increase of equine cases of infection developed as a mechanism for viral survival [43]. Accordingly, an increment of the bat population near urban environments magnifies the interaction rate with horses and people. Considering the role that bats hold as reservoirs of a large number of virus families, this implies a higher risk for the emergence of viral diseases. Likewise, animals currently classified as Endangered Species due to human activity tend to share more viruses with humans in comparison to species endangered caused by other reasons, showing a strong correlation between conservation state and the presence and possible zoonotic transmission. Domestic animals and wildlife species have common viruses suggesting that the original hosts have been long located in the wild, thus, the detection of these viruses in domesticated animals is the result of coevolution with domestic practice [26]. Hence, both direct and indirect contact with wildlife for handling, ecotourism, or investigational purposes, propose a risk for zoonotic circulation. 

Another factor known to affect the transmissibility of viruses to the human population is cultural practices [43]. Changes in farming and food consumption patterns increase the rate of interactions between wildlife and humans. For example, the use of wild animals as food near wild habitats are said to increase the probability of pandemic emergence [6,43]. In Asia, live markets are used for trading wild animals such as squirrels, rats, porcupines, as well as wild birds and pigs. These animals are sold in many ways (alive, dead, or in pieces) to consumers. This distribution process typically involves at least three people: hunter, seller, and consumer; a fact with huge epidemiological implications for viral transmission. It has been identified that the main reasons for wild animal consumption are food source and, to a lesser extent for medicinal purposes [24].

Pandemic control caused by zoonotic potential viruses strongly depends on human lifestyle and awareness about the existence of such diseases and the probability to acquire them [44]. Although people tend to acknowledge zoonotic diseases and their sources, the risk of getting infected is not well dimensioned. Domestic animals are mostly associated with influenza, rabies, coccidiosis, and brucellosis, while diseases such as HIV, leptospirosis, and dengue are mostly related to wild animals [24,45,46].

The occurrence of epidemics and pandemics during the last decade makes evident the importance of airports for disease transmission. As seen with SARS-CoV-1 in 2003, late responses in Hong Kong and Singapore airports due to unpreparedness for an emergency of such magnitude, made propagation of the disease easier around China and in some other Asian countries [34]. Further controls and strategies are required to help identify infected people and improve the contention of disease outbreaks through airports. Given the unprecedented speed, volume, and extent of travel and international trade, the association between transmission by spreaders to the general population and long-distance movements is undeniable. Long-distance travel and trades increase the contact rate between animal reservoirs and humans [47]. Limiting human contact is often difficult, ergo restricting the scale of legal and illegal movements of domestic animals and wildlife species presents a real opportunity to minimize emergence risks [48,49]. 

### 3.3. Case Study: Influenza Virus in Latin America

The importance to increase surveillance of viruses such as influenza-, corona-, and hanta- viruses, among other infectious agents of great importance to public health in Latin America, is well emphasized in several articles [50,51,52,53,54,55]. In 2015, influenza was considered the disease with the best simulation exercise completion index and nearly 85% of LAC said to have up-to-date contingency plans in case an emergency occurs. Even though, nearly 60% of LAC have a Memorandum of Understanding between governments, most countries agree that for Influenza the most important capacities to improve are diagnosis, surveillance, and coordination among ministries, universities, ONGs, the private sector, and central or local authorities in the community. This governmental interest to obtain “reliable local surveillance data” to improve current prevention strategies substantially increased since the 2009 H1N1 pandemic, as most vaccine policies are based on global standards. The H1N1 pandemic helped to raise awareness of the impact of prevention activities against the disease, hence, the incorporation of genetic sequencing technology and techniques have been essential toward understanding the genetic evolution of influenza virus. Although 71% of health and agricultural ministries consider moderate to probable the introduction of Influenza into their countries, additional intervention is needed from the governmental standpoint [46]. 

Within Latin America, there are currently 22 laboratories designated as National Influenza Centers (NICs) and one WHO Collaborating Center (CC) for Influenza Surveillance (U.S. Centers for Disease Control and Prevention (U.S. CDC)), part of the Global Influenza Surveillance and Response System (GISRS). These labs provide information about genetic evolution and patterns for Influenza A and B viruses. Data obtained from 2010–2018 have shown that vaccination plans in LAC tend to correlate with genetic findings of circulating influenza virus in the region, although the characteristics of seasonal subtype of the virus are unevenly reported across countries [50,54,56].

#### 3.3.1. Factors Associated with the Virus and its Hosts

Spatio-temporal patterns of influenza epidemics in Latin America are highly variable and depend on the genetic characterization of the virus, the distribution, behavior and ecology of the host, and other environmental, climatologic, and politically related factors. Among the research articles reviewed conducted in LAC, factors that impact the transmissibility of the virus associated with the characterization of the viral genome were mentioned in almost half of the papers (*n* = 8). Even though good surveillance data exists, the characterization of the viral genome is a common gap reported in most LAC. Starting in 2009, all NIC labs have implemented the use of a combination of indirect immunofluorescence and qRT-PCR to test for influenza virus. Seasonal dynamics and genetic evolution studies have shown that the H3N2 virus remained the dominant FluA subtype between 2000 and 2017, except during 2009, when H1N1pdm was the most diagnosed subtype. It is worth mentioning that despite the entrance of the pandemic strain, the seasonal subtype H3N2 was diagnosticated at a normal rate. During this period, FluB virus was attributed to almost 25% of all Flu reported cases, and subtypes Yamagata and Victoria were equally reported displaying no dominance between them, although it was until 2013 that the WHO and CDC provided kits and training for FluB lineage testing [50,51,54]. 

The H1N1pdm virus was assigned to nearly 50% of all cases reported in the first 15 years of the 21st century in the region, even though most of them took place in 2009. Records show that pandemic cases were more frequent in patients younger than 20 years, although patients between 50–59 were prone to register the highest viral loads and mortality rate [50,51]. This public health emergency enhanced the collective action of vaccination programs across LAC. As of 2017, 38 out of 50 LAC reported having a vaccine policy for the annual use of seasonal Influenza vaccine, from which 90% of the countries use trivalent vaccines. Trivalent vaccines protect against two influenza A viruses and one lineage of influenza B viruses. Based on the predominant clades for H1pdm and FluB viruses found in human samples between 2000 and 2017 from 34 Latin American and Caribbean countries, 79% of all seasons had a predominant lineage and in 32% there was a mismatch with the currently available vaccine [54]. The year-to-year evolution of circulating influenza viruses is certainly the biggest obstacle for control, thus continuous diagnosis and sample sequencing is key to update running vaccination plans. Between 2017 and 2018, 12 NICs reported that results from samples taken in LAC matched global patterns and were fully covered by the vaccine recommended virus; even though H3 clades not associated with the vaccine recommended virus were found, studies have shown cross-reaction using trivalent vaccine viruses against this HA [56]. 

The genetic diversity of influenza viruses in Latin America is evident when the variety of hosts that support their survival is taken into account. Wild birds, pigs, and bats are cataloged as natural reservoirs for influenza viruses [18,52,57,58]. Positive samples obtained from aquatic wild birds in Peru between 2006–2011 showed that most sequences were related to North American lineages and that findings were consistent with regular migration patterns from these wild birds. Interestingly though, an unusual reassortment of an H13 virus containing a PA segment related to remarkably divergent Argentinian H6 viruses, leads to believe that substantial influenza virus A diversity circulates undetected throughout South America; this suggests that the evolution of independent lineages in LAC is possible and that the introduction and amplification of the virus could occur in mixing grounds where native and migratory species interact [18,52]. H1N1pdm virus reassortments with human-like SIV (Swine Influenza Viruses) found in pig farms with respiratory signs, were initially reported in 2011 in Argentina, showing the constant evolution of influenza viruses at the human–swine interface. Rural studies that evaluate animal husbandry practices in Ecuador revealed that pigs represent 60% of the animals raised as backyard herds, magnifying the risk for zoonotic diseases from this natural host [44,57].

Bats, on the other hand, due to their flying capacity, long migrations, trophic diversity, and social structure, have an extraordinary viral richness. H18 Influenza viruses reported in fruit bats in Peru show that the coevolutionary process has occurred for a long time based on the genetic diversity found within the gene segments, the use of alternative mechanisms to enter the host cells, and the broad geographic distribution, after comparing it to bat seroprevalence data obtained in Central American Countries [58]. The social structure of these flying mammals involves a dense network and widespread allocation due to high adaptability to ecologic and environmental changes. Bats have been located near human settlements across the Amazonian region proposing a huge threat of zoonotic transmission not only for influenza virus but also for high-risk viruses such as hantaviruses [18,59].

Rodents have been recognized as reservoirs for several zoonotic viruses and even though no reports of influenza viruses have been found in this animal, the closely related host-to-host network with bats suggests a possible exchange of other high-risk zoonotic pathogens [18,60]. It is valuable to note that hantaviruses have been used to evaluate the impact of rodents as reservoirs of agents of great importance for public health broadly distributed around the globe. In LAC, Hantavirus Pulmonary Syndrome represents a major emerging disease of the last century and is expected to remain a public health threat in the future. Considering the ecology and behavior of rodents in different LAC, the spillover effect result of the overlap in habitat and food sharing among different species implies the finding of viruses in uncommon hosts and suggests the easy transmission of virological strains in the region [53,61,62,63].

#### 3.3.2. Other Factors That Affect the Interspecies Transmission

Other factors that affect the transmissibility of influenza viruses and other RNA viruses of important public health risk are closely related to the environment, the climatology, and the politics of LAC. The seasonal characteristics of Influenza A and B epidemics show a distinctive pattern when latitude is considered. Studies developed in Brazil have demonstrated that cases of Syndrome of Acute Respiratory Disease that are related to influenza virus show a tendency similar to a wave coming from northern latitudes with a delayed peak as it moves south the continent [55]. This wave is analogously described for pneumonia and mortality rates related to influenza infection, and records prove a one-month lag between viral isolation and mortality. In northern Brazilian tropical regions, mortality peaks tend to occur during April and May. Meanwhile, southern subtropical states are the most affected territories, and during the winter months, Influenza infection is more prevalent [64]. Differences between tropical and subtropical territories show that LAC closer to the Ecuadorian Line have low primary peaks of infection but multiple secondary peaks throughout the year [50]. FluB circulation patterns reviewed between 2010 and 2017 in LAC show similarity to FluA seasonality. In Andean and Central American countries FluB was identified year-round, while in the temperate southern cone it was detected at the end of Flu seasons [54].

Environmental factors have shown to have a bigger impact on disease dynamics when compared to demographics, traveling, and other human population indexes. Even though rainfall and low temperatures are associated with influenza seasonality, these seem to be more related to human behavior by increasing human proximity and by developing higher physiological stress levels that could lead to infection [64]. Several anthropogenic factors enhance viral transmission and pathogenicity. Probability maps have shown that rural territories with a high probability of contact with wild animals tend to have the highest number of zoonotic cases and also the highest probability of recurrence [53]. Amazonian wild areas that have been prominently affected by human intervention for agricultural practices, farming, and logging, have brought natural reservoirs like rodents, bats, primates, and mosquitoes, along with zoonotic viruses closer to human settlements [44,61]. Vaccination plans in marginal zones are low to null although more consideration is being taken from Health and Agricultural Ministries for endemic and emerging zoonotic diseases. The improvement of the health coverage in LAC is key to avoid the spread of zoonotic viruses to more populated areas [46,54,65].

## 4. Discussion

The infectious capacity of the agent and the susceptibility of the host are derived from a range of factors that act in a synergic, mitigating, or amplifying manner. Although the recognition of factors associated with RNA viruses and their relationship with the host, such as the affinity or preference of binding to specific receptors, is evident, the success for viral replication requires the virus to adopt mechanisms for the evasion of immune response. Moreover, the establishment of a disease in a susceptible population will also be determined by processes that involve environmental, anthropogenic, and ecological factors.

According to the results obtained in our review, changes at the molecular level do not necessarily have to be of great magnitude to have a high influence on the manipulation of the innate defense systems of the infected cell. As an example, the TC dinucleotide is one of the preferred target sequences of the host restriction factor (APOBEC3 proteins), thus, the suppression of this dinucleotide could be the result of an evolutionary selection that allows coronaviruses to evade the host’s immune protection capacity [15]. Susceptibility to certain viruses due to the presence of the receptor is an aspect that must be considered when evaluating the probability of the occurrence of jumps between species. The use of host cell receptors that are highly conserved across different species has been suggested to facilitate infection in a wide range of hosts. This may explain the circulation of the virus among various types of hosts and the easiness for human infection.

Before the SARS-CoV-2 emergency, interest and concern for coronavirus infection in humans was limited, and much of the knowledge and information available came from studies and research on diseases in veterinary medicine. The applicability of this knowledge in isolation and diagnosis contributed to achieving an accelerated international response to the pandemic, as well as to understanding the aspects related to pathogenesis and immune response. Intrinsic factors and host-related characteristics also need to be investigated in order to adopt control measures in addition to the ones aimed specifically against the pathogen. The genetic basis for disease resistance is that the inherent genetic diversity of the population provides a wide range of possibilities for pathogens in their evolutionary process to acquire capacities to evade the immune response. Hence, when a virus sets in motion a strategy to evade an individual’s immune defenses, the genetically diverse population may still be protected. However, once the pathogen manages to establish an infectious process in a host, the necessary conditions for consequent transmission, pathogenesis, and morbidity for the rest of the population due to genetic and immunological homogeneity are reshaped.

The level of awareness in communities about viral diseases transmitted between humans and animals has been an important detail reported in many countries. Finding positive results in terms of the description of specific diseases, reservoirs, symptoms, and transmission routes shows that it is not an unknown subject. Cultural characteristics and social beliefs have a strong influence on the consumption, handling, sale, and hunting of wild animals when compared to the level of education existing in the population. People who access animal markets are of extremely diverse socioeconomic traits, ranging from farmers to public servants. The preference in the consumption of certain species of animals supports the conclusion that the species that are threatened by human causes are those most implicated in the emergence of pandemic viruses [24]. 

The role of domestic animals in the transmission and emergence of viral diseases has resulted in the increasingly efficient implementation of biosecurity practices for the prevention of diseases that affect animal production and for those that can give rise to large pandemics. Approaches such as maintaining a clean and disinfected environment through physical decontamination, the slaughter of infected animals, the decontamination of vehicles, equipment, and restriction of people moving to and from the site, and preventive vaccination are important to a comprehensive approach to biosecurity. Animal healthcare practices in LAC are poorly emphasized in rural areas. Even though backyard livestock production has been the focus of many development and antipoverty programs, efforts to improve animal sanitation have been proposed as promising interventions to downgrade the impact of farming practices on viral and other pathogens transmission. Thanks to this knowledge, the devastating effects of a pandemic such as the high mortality of the susceptible population, high rates of contagion, or consequences associated with the social and economic sphere require the implementation of control measures to a greater extent.

Based on the information gathered from this review, bats in many parts of the world including LAC are known to harbor high levels of virus diversity. The wide distribution of their habitats with an impressive geographic range with different levels of prevalence in almost all the investigated species supports the important role they have in the evolution of these viruses. However, the lack of congruence of some data has allowed, to a certain degree, to know about the close phylogenetic relationship of coronavirus of bats of the same genus with different species of coronavirus that tend to be associated with other genera of bats, suggesting multiple introductions of coronaviruses in these animals. Multiple introductions into *Rhinopomatidae* bats suggest that interspecies transmission events may be enhanced by sharing roosting sites with different species, including *Myotis, Miniopterus,* and *Rousettus* bats. In the Latin American context, this type of information is scarce, and therefore the implementation of new studies to identify the bat species involved as reservoirs of different types of viruses, their ecology, and the interaction they may have with other congeners is necessary to better understand the epidemiology of diseases originating in these species with the potential to make jumps to humans.

Research about the Latin American setting has been mainly conducted by institutions and universities located outside the region. The reduced number of publications in Latin American journals manifest the reduced impact of such platforms, calling for action to further investigate the circumstances that could present a big risk for the development of highly impactful diseases in LAC. Based on the cut-off date used for this review, the Latin American case study only included influenza-related data since databases did not show SARS-CoV-2 studies in the region enough to make an analysis by the time this review was conducted.

The growth rate and the magnitude of the human and animal circulation added to climate change create a unique global situation that favors contact between different populations, constituting a real zoonotic risk. Human migration from rural to urban and peri-urban areas is associated with a high risk for disease transmission due to generally poor living conditions, shared living spaces, and competition for resources. In LAC, this association is also seen for human migration at a country level, as multiple social and economic issues have encouraged the movement of people outside their homeland in search for a better life; an ongoing example is the massive exodus from Venezuela to many countries in the region, that has been associated with the increase of cases of Malaria [66]. Therefore, it is necessary to determine the impact that human migration in LAC could have in spread of viral infections as well. Although environmental constituents tend to have a more substantial impact than social determinants, political factors associated with health coverage and disease prevention display a major disadvantage to thoroughly prepare and eventually outlive a possible emergence of a zoonotic disease of pandemic potential in the region. Despite efforts made among Amazonian countries to prevent the environmental crisis, such as the “Leticia Pact for the Amazon” celebrated in September 2019 [67], there are still no intervention policies that involve the community health component. A prediction study that evaluated the relationship between the international indicators Global Environmental Stratification (climate) and mammal species richness (an aspect of biodiversity) with the presentation of emerging zoonotic diseases, suggested a higher risk in tropical forest regions, associated with greater biodiversity and land use changes [68]. Remarkably, the areas with the highest probability of these phenomena occurring are where the indigenous communities and the poorest rural populations in the world are concentrated. Moreover, the abundant biodiversity in the Amazonian territory in addition to the passing of migratory routes of wild animals promotes the interaction between humans and wildlife, illustrating the relevance to further investigate the region and fill in the gaps of information currently identified. 

Recognizing the importance of social and environmental impact to the emergence of viral diseases is fundamental. In the same context, it is essential to identify the connections between these factors and their effect on the dynamics of diseases to mitigate negative outcomes to the human population. The understanding of the different factors that intervene in the appearance of viral agents with pandemic potential seeks to provide arguments that contribute to the preparation, timely detection, and prompt response to the imminent appearance of agents with characteristics that pose a threat to health and the global economy. Therefore, it is essential to study and conduct research, not only regarding viral but also social, cultural, and anthropogenic factors that could favor the establishment and appearance of viruses with epidemic and/or pandemic potential in LAC.
